# Alpha B-crystallin is a new prognostic marker for laryngeal squamous cell carcinoma

**DOI:** 10.1186/1756-9966-31-101

**Published:** 2012-12-12

**Authors:** Yuan Mao, Da-Wei Zhang, Hong Lin, Lin Xiong, Ying Liu, Qing-Dong Li, Jun Ma, Qing Cao, Ren-Jie Chen, Jin Zhu, Zhen-Qing Feng

**Affiliations:** 1 Department of Otolaryngology-Head and Neck Surgery, Jiangsu Province Official Hospital, No.65 Jiangsu Road, Nanjing 210029, China; 2 Department of Otolaryngology-Head and Neck Surgery, The Second Affiliated Hospital of Nanjing Medical University, No.121 Jiang jia yuan, Nanjing 210011, China; 3 Jiangsu Provincial Blood Center, No.179 Longpan Road, Nanjing 210042, China; 4 Department of Pathology, The Second Affiliated Hospital of Nanjing Medical University, No.121 Jiang jia yuan, Nanjing 210011, China; 5 Department of Otolaryngology-Head and Neck Surgery, Yiji Shan Hospital of Wannan Medical College, No.92 Zheshan Xi Road, Wuhu 241000, China; 6 The Key Laboratory of Cancer Biomarkers, Prevention & Treatment Cancer Center and The Key Laboratory of Antibody Technique of Ministry of Health, Nanjing Medical University, No.140 Hanzhong Road, Nanjing 210029, China; 7 Huadong Medical Institute of Biotechnology, No.293 Zhongshan Dong Road, Nanjing 210002, China

**Keywords:** Laryngeal carcinoma, Alpha B-crystallin gene, RT-PCR, qPCR, Immunohistochemistry, Prognosis

## Abstract

**Background:**

Alpha B-crystallin (αB-crystallin) has been suggested to play an important role in the development of solid tumors. However, the association between αB-crystallin expression and clinicopathological characteristics of human laryngeal carcinoma is not well defined. This study aimed to examine the expression of αB-crystallin in human laryngeal squamous cell carcinoma (LSCC) and investigate the relationship between its expression and the prognosis of LSCC.

**Methods:**

Real-time polymerase chain reaction (six LSCC samples, six tumor-adjacent normal samples) and immunohistochemistry by tissue microarrays (109 LSCC samples and 28 tumor-adjacent normal samples) were performed to characterize expression of the αB-crystallin gene in LSCC. Kaplan-Meier survival and Cox regression analyses were carried out to evaluate the prognosis of LSCC.

**Results:**

Real-time polymerase chain reaction and immunohistochemistry analysis showed that the expression of αB-crystallin in LSCC was significantly higher than that in tumor-adjacent normal tissues. Moreover, the expression level of αB-crystallin protein in LSCC was significantly related to alcohol consumption (P = 0.022), tumor differentiation (P = 0.007), pTNM stage (P = 0.041) and 5 years’ survival (P =0.030). COX multi-factor analysis showed that αB-crystallin (P = 0.013), as well as pTNM stage (P =0.027) and lymphatic metastasis (P = 0.015) were independent prognosis factors for LSCC.

**Conclusions:**

The data suggest that αB-crystallin expression is correlated with malignant phenotypes of LSCC and it may serve as a novel prognostic factor for LSCC.

## Introduction

Squamous cell carcinoma (SCC) of the head and neck is one of the most frequent malignancies in the world, with about a quarter of all cases occurring in the developing countries. SCC accounts for nearly 90% of all head and neck carcinomas [1]. Approximately, one-fourth of all head and neck cancers are laryngeal squamous cell carcinoma (LSCC). LSCC is a malignant tumor of laryngeal epithelial origin and the clinical symptoms usually depend on its original site and size [2,3]. Although several cutting-edge treatment strategies have been developed for LSCC, no treatment could achieve a satisfactory therapeutic outcome and the mortality rate of LSCC is still high (5-year survival rate is 64%) [4]. Therefore, it is urgent to develop novel and valuable markers to distinguish patients with poor prognosis or at high risk of early recurrence and guide chemotherapy and radiotherapy [5].

Alpha B-crystallin (αB-crystallin) is a member of the small heat shock protein (sHSP) family and acts as a molecular chaperone, by preventing the aggregation of denatured proteins after the exposure to stresses such as heat shock, radiation, oxidative stress and anticancer drugs [6]. Moreover, ectopic expression of αB-crystallin in diverse cell types confers protection against a variety of apoptotic stimuli, including TNF-α, TNF-related apoptosis-inducing ligand (TRAIL), etoposide and growth factor deprivation [7,8]. It is believed that αB-crystallin can interact with different apoptotic proteins to regulate apoptosis [9]. Recent studies suggest that αB-crystallin is a prognostic marker for various types of solid tumors [10-12]. αB-crystallin may play a role in tumorigenesis by modulating vascular endothelial growth factor (VEGF) [13,14]. However, the expression and function of αB-crystallin in LSCC have not been determined.

In this study, we examined the expression levels of αB-crystallin in LSCC tissues and tumor-adjacent normal tissues. The results showed that compared to tumor-adjacent normal tissues, αB-crystallin expression was higher in LSCC tissues. In addition, αB-crystallin expression in LSCC was associated with alcohol consumption, tumor differentiation, pTNM stage and 5-year survival.

## Materials and methods

### Patient specimens

A total of one hundred and nine cases of LSCC were collected from the Department of Pathology, the Affiliated Hospital of Nantong University between 2000 and 2009. Diagnosis of LSCC was determined according to the latest WHO criteria [15] and TNM stage classification (UICC 2002). Among the cases, there were 107 men and 2 women. The mean age of patients at the time of surgery was 60.8 years (ranging from 29 to 87 years). Related clinical data were collected, including gender, age, tobacco and alcohol consumption, tumor differentiation, pTNM stage, lymph node metastasis, and 5-year follow-up survival. Follow-up in all patients started from post-operation of May 2010. None of the 109 patients had performed radiotherapy, chemotherapy or immunotherapy before the surgery. Study protocol was approved by the Ethics Committee of Jiangsu Province Official Hospital.

### Reverse transcription-polymerase chain reaction (RT-PCR) and quantitative real-time polymerase chain reaction analysis (qPCR)

Six samples of fresh LSCC tissues and their adjacent tissues were collected from the Department of Otolaryngology-Head and Neck Surgery, the Second Affiliated Hospital of Nanjing Medical University and the Department of Otolaryngology-Head and Neck Surgery, Yiji Shan Hospital of Wannan Medical College. Total RNA was extracted from LSCC tissues and tumor-adjacent tissues by using Trizol reagent (Invitrogen, Carlsbad, CA) according to the manufacturer's protocol. RNA (2 μg) was reverse transcribed using High-Capacity cDNA Archive Kit (Promega) in accordance with the manufacturer's protocols. Primers were as follows: αB-crystallin forward 5’-CTTTGACCAGTTCTTCGGAG-3’, reverse 5’-CCTCAATCACATCTCCCAAC-3’; β-actin forward 5’- CTCCATCCTGGCCTCGCTGT-3’, reverse 5’- GCTGCTACCTTCACCGTTCC-3’. The transcription levels of β-actin served as a loading control. Analysis of qPCR was performed using SYBR green dye in an ABI PRISM 7000HT Sequence Detection System (Applied Biosystems, Foster City, CA, USA) following the manufacturer’s instructions. Cycle conditions were as follows: after an initial incubation at 50°C for 2-min and at 95°C for 10 min, the samples were cycled 40 times at 95°C for 15 seconds and 56°C for 1 min.

### Tissue microarrays (TMA) construction and immunohistochemistry

Formalin-fixed, paraffin-embedded tissues from 109 LSCC and 28 tumor-adjacent normal tissues were prepared and utilized in this present study. TMA was produced by Xinchao Biotech (Shanghai, China). Core tissue biopsies (2 mm in diameter) were taken from individual paraffin-embedded LSCC and arranged in the new recipient paraffin blocks. Tissue microarray was cut into 4-μm sections and placed on super frost charged glass microscope slides.

Paraffin tissue sections (4 μm) were deparaffinized in 100% xylene and re-hydrated in descending ethanol series and water according to standard protocols. Heat-induced antigen retrieval was performed in 10 mM citrate buffer for 2 min at 100°C. Endogenous peroxidase activity was blocked by hydrogen peroxidase (3%) in Tris-buffered saline (TBS) for 30 min. Then the sections were boiled for 10 min in citrate buffer for antigen retrieval. Nonspecific binding was blocked by incubation with 5% goat serum in TBS for 30 min. Tissue sections were incubated with mouse anti-αB-crystallin antibody (Stressgen, Victoria, Canada; 1:300) in TBS containing 1% bovine serum albumin for 1 h. After washing, sections were incubated with EnVision goat anti-mouse/horseradish peroxidase antibody (EB-2305, ZhongShan, Godbridge, China; 1:2000) for 1 h. The replacement of the primary antibody with PBS served as negative controls. Finally, the sections were developed with 3,3-diaminobenzidine (DAB) chromogen solution and counterstained with hematoxylin. Four fields in each slide were randomly selected and counted, and the percentage of positive staining was determined by two clinical pathologists independently using immunohistochemistry score (IHS) [16]. When a conclusion differed, the final decision was made by consensus. The results were analyzed according to the method described previously [17]. Briefly, IHS was determined by the evaluation of both staining density and intensity. The percentage of positive tumor cells was scored as follows: 1 (0-10% positive cells), 2 (11-50% positive cells), 3 (51-80% positive cells), 4 (81-100% positive cells); and the intensity of staining was scored as follows: 0 (negative), 1 (weakly positive), 2 (moderately positive), and 3 (strongly positive). Multiplication of the intensity and the percentage scores gave rise to the ultimate IHS: a sum score below 3 indicated low expression of αB-crystallin, and a sum score above 4 indicated high expression of αB-crystallin.

### Statistical analysis

The relationship between αB-crystallin expression and clinicopathological factors was analyzed by chi-square test. Survival rate was estimated by Kaplan-Meier method. Univariate and multivariate analysis was carried out using Cox’s proportional hazards regression models. For all tests, the significance level for statistical analysis was set at P < 0.05. Statistical analyses were performed using STATA Version 12.0 (Stata Corporation, College Station, TX).

## Result

### High expression of αB-crystallin mRNA in LSCC

**Figure 1:**
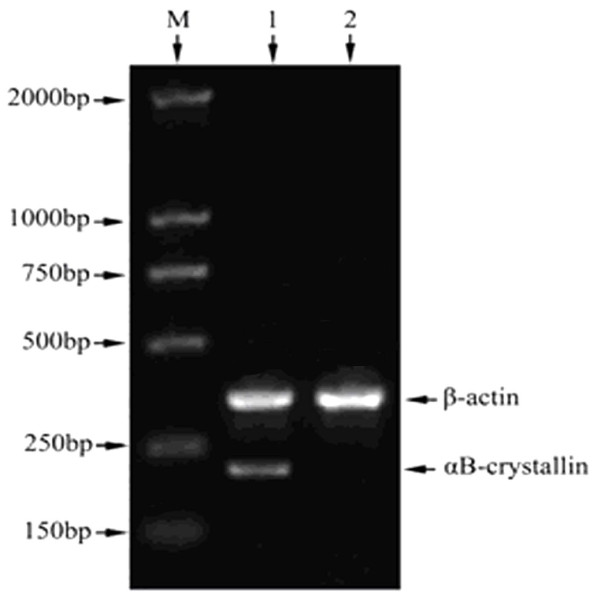
**Detection of αB-crystallin mRNA expression in LSCC tissue and normal tumor-adjacent tissue.** Line M: DNA marker (DL2000, TAKALA, Dalian, China); line 1: LSCC tissues; line 2: normal tumor-adjacent tissues. Shown were representative images from three independent experiments.

**Figure 2:**
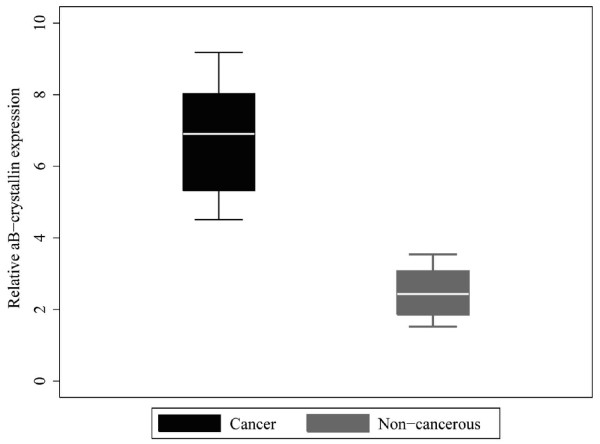
**The mRNA levels of αB-crystallin determined by qPCR.** The relative mRNA level of αB-crystallin was higher in LSCC than in normal tumor-adjacent tissue (p < 0.05).

RT-PCR amplicons were detected by 1.5% agarose gel electrophoresis, confirming that αB-crystallin was expressed in LSCC tissues (Figure 1). Moreover, mRNA levels of αB-crystallin in LSCC tissues and tumor-adjacent tissues were determined by qPCR. Normalized to β-actin, αB-crystallin mRNA level in LSCC tissues (n = 6) and tumor-adjacent normal tissues (n = 6) was 6.808 ± 1.781 and 2.475 ± 0.757, respectively (t = 5.484, P = 0.001). mRNA level of αB-crystallin was significantly higher in LSCC than in tumor-adjacent normal tissues (Figure 2).

### αB-crystallin protein level is correlated with the clinicopathologic factors of LSCC

**Figure 3:**
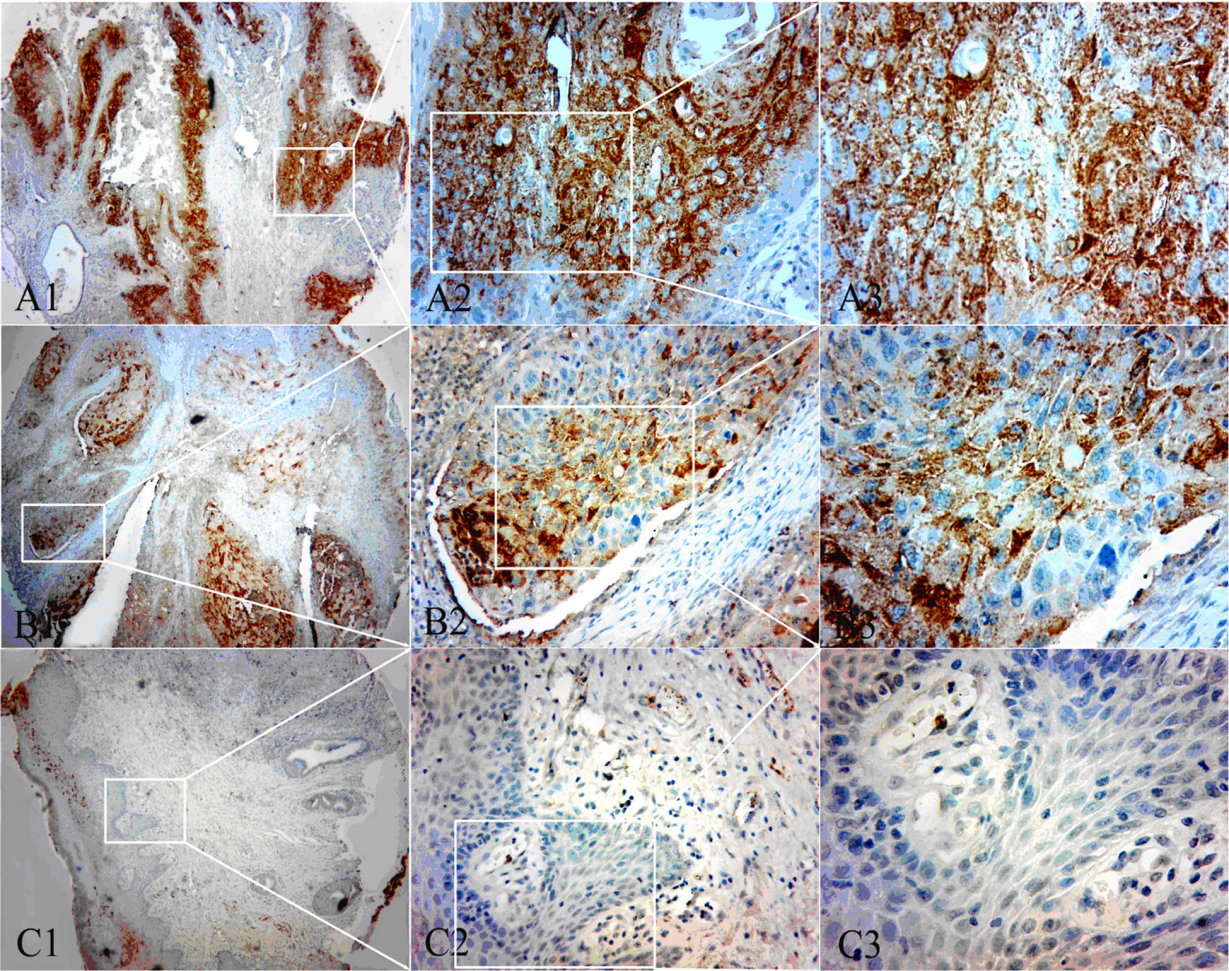
**Expression pattern of αB-crystallin in tumor tissue and tumor-adjacent tissue of LSCC.** TMA sections were analyzed by immunohistochemical staining. Brown staining indicated positive expression of αB-crystallin. A1-3: The expression pattern of αB-crystallin in moderately differentiated LSCC tissue. B1-3: The expression pattern of αB-crystallin in well-differentiated LSCC tissue. C1-2: The expression pattern of αB-crystallin in tumor-adjacent tissue with weakly positive staining of αB-crystallin. C3: Squamous epithelium of adjacent nontumorous tissue with negative staining of αB-crystallin. Original magnification: ×40 in A1, B1 and C1; ×100 in A2, B2 and C2; ×400 in A3, B3 and C3.

By immunohistochemistry analysis, we observed more positive staining cells and stronger staining in LSCC tissues than in tumor-adjacent normal tissues (Figure 3). The positive staining was localized mainly in the cytoplasm of the tumor cells and strong staining was not observed in the surrounding tumor-adjacent areas. Positive staining of αB-crystallin was detected in 64 (58.72%) of 109 LSCC samples, while only 5 cases of 28 tumor-adjacent normal tissues (17.86%) displayed high expression of αB-crystallin. There was significant difference in high expression rate of αB-crystallin between LSCC tissues and normal non-cancerous tissues (P = 0.001).

Correlations between various clinicopathological characteristics and αB-crystallin expression in LSCC tissues were evaluated by χ^2^ test (Table 1). The result showed that high expression of αB-crystallin in LSCC was significantly related to alcohol consumption (P = 0.022), tumor differentiation (P = 0.007), pTNM stage (P = 0.041) and 5-year survival (P = 0.030). However, no statistically significant correlation was found between αB-crystallin expression and gender, age, tobacco use, or lymph node metastasis.

**Table 1:** Correlation of aB-crystallin expression with clinicopathological characteristics of LSCC

**Groups**	**No.**	**aB-crystallin**		**χ**^**2**^	**P (value)**
		**+**	**%**		
Gender					
Male	107	63	58.88	0.0638	0.801
Female	2	1	50.00		
Age(years)					
≤60 y	45	23	51.11	1.8283	0.176
>60 y	64	41	64.06		
Tobacco use					
Yes	77	42	54.55	1.8816	0.170
No	32	22	68.75		
Alcohol consumption					
Yes	53	37	69.81	5.2395	0.022^*^
No	56	27	48.21		
Tumor differentiation					
Well	51	22	43.14	9.9434	0.007^*^
Moderate	53	39	71.70		
Poor	5	3	80.00		
pTNM stage					
Stage I, II	65	33	50.77	4.1945	0.041^*^
Stage III, IV	44	31	70.45		
Lymph node metastasis					
Yes	19	14	73.68	2.1270	0.145
No	90	50	55.56		
Five years’ survival					
Yes	72	37	51.39	4.6972	0.030^*^
No	37	27	72.98		

* P < 0.05.

### Survival analysis

**Figure 4:**
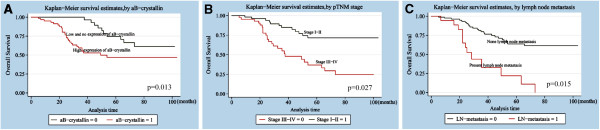
**Survival curves of LSCC patients based on various independent factors.A**: Overall survival rate in patients with positive expression of αB-crystallin (red line, αB-crystallin = 1) was significantly lower than that in patients with negative αB-crystallin expression (green line, αB-crystallin = 0). **B**: Overall survival rate in patients with stage III-IV of LSCC (red line, stage III-IV = 0) was significantly lower than that in patients with stage I-II of LSCC (green line, stage I-II = 1). **C**: Overall survival rate in patients with lymph node metastasis (red line, LN metastasis = 1) was significantly lower than that in patients without lymph node metastasis (green line, LN metastasis = 0).

Univariate analysis showed that the life span of LSCC patients was correlated with αB-crystallin expression (P = 0.010), pTNM stage (P < 0.001), lymph node metastasis (P < 0.001) and tumor differentiation (P = 0.022). Multivariate analysis with the Cox regression model indicated that αB-crystallin protein level may serve as an independent prognostic factor for overall survival (P = 0.013) (Table 2). Furthermore, pTNM stage (P = 0.027) and lymph node metastasis (P = 0.015) were identified as independent predictive factors for poor outcome of LSCC. Kaplan-Meier survival curves showed that patients with high αB-crystallin expression had a shorter survival time than patients with low αB-crystallin expression (Figure 4). Kaplan-Meier survival curves demonstrated that patients with high αB-crystallin expression, advanced pTNM stage of LSCC and lymph node metastasis had a significantly shorter survival time.

**Table 2:** Univariate and multivariable analysis of prognostic factors in LSCC for 5-year survival

	**Univariate analysis**			**Multivariable analysis**		
	**HR**	**p > |z|**	**95% CI**	**HR**	**p > |z|**	**95% CI**
αB-crystallin expression						
High versus Low	2.508	0.010^*^	1.245-5.051	2.498	0.013^*^	1.218-5.124
Age (years)						
≤60y versus >60y	0.613	0.148	0.316-1.189			
Tobacco use						
Yes versus No	0.643	0.203	0.325-1.270			
Alcohol consumption						
Yes versus No	0.903	0.747	0.485-1.680			
pTNM stage						
Stage I, II versus Stage III, IV	0.291	0.001^*^	0.151-0.561	0.426	0.027^*^	0.200-0.908
Lymph node metastasis						
Yes versus No	4.412	0.001^*^	2.225-8.748	2.707	0.015^*^	1.215-6.034
Tumor differentiation						
Well versus Moderate-Poor	0.478	0.022^*^	0.255-0.897	0.594	0.107	0.315-1.120

* P < 0.05.

## Discussion

Several state-of-the-art treatment strategies have been developed for LSCC, including molecular targeted therapy [18], gene therapy [19] and immunotherapy [20]. However, no treatment could achieve satisfactory therapeutic outcome and the survival rate of LSCC has not been improved significantly [21]. Recent studies suggest several molecular markers of LSCC [22-24]. Further identification of prognostic markers for LSCC will be important for the prevention and therapy of LSCC.

αB-crystallin has been shown to be overexpressed in numerous kinds of tumors, including gliomas, prostate cancer, oral squamous cell carcinomas, renal cell carcinomas, and head and neck cancer [25]. Recently, an oncogenic role of αB-crystallin has been proposed for breast cancer [26]. The neoplastic changes and invasive phenotypes of breast cells and the anti-apopototic activities of αB-crystallin were inhibited by the phosphorylation of αB-crystallin [27,28]. Furthermore, αB-crystallin could promote tumor angiogenesis by modulating VEGF [13,14]. These studies demonstrate that αB-crystallin plays crucial role in tumor progression.

In the present study, the mRNA and protein levels of αB-crystallin in LSCC and tumor-adjacent normal tissues were detected by qPCR and immunohistochemistry. Both analyses showed that αB-crystallin was highly expressed in LSCC compared to tumor-adjacent normal tissues. These results agree with previous report which showed that αB-crystallin was overexpressed in hepatocellular carcinoma cells compared with non-tumour cells [11]. Moreover, we found that the high expression of αB-crystallin in LSCC was related to alcohol consumption, tumor differentiation, pTNM stage and 5-year survival.

Univariate analysis showed that not only αB-crystallin expression, but also the pTNM stage, lymph node metastasis and tumor differentiation were correlated with life span of LSCC patients. Multivariate analysis revealed that strong expression of αB-crystallin could be considered as an independent factor for poor prognosis of LSCC patients, as well as pTNM stage and lymph node metastasis.

Interestingly, several studies suggest that αB-crystallin acts as a tumor suppressor gene in certain types of cancer [29-31]. In addition, αB-crystallin staining was reported to be reduced in head and neck squamous cell carcinoma and αB-crystallin was not proposed as a prognostic marker [32,33]. Our present data are inconsistent with these studies. These conflicting results may be due to the differences in the pathological samples, the antibodies used, the experimental methods or evaluation system.

In conclusion, to the best of our knowledge, this is the first study to report that high αB-crystallin expression is correlated with aggressive malignant phenotype of LSCC. Our data indicate that αB-crystallin may serve as a novel prognostic marker for LSCC. Further studies are needed to confirm the prognostic and therapeutic value of αB-crystallin for LSCC.

## Conclusions

Taken together, the results of this study suggest that αB-crystallin expression is correlated with malignant phenotypes of LSCC and it may serve as a novel prognostic factor for LSCC.

## Competing interests

The authors declared that they have no competing interest.

## Authors’ contributions

YM and DWZ design the study; HL, YL and QDL carried out the RT-PCR and qPCR analysis; LX, JM and QC peformed the immunohistochemistry; YM drafted the manuscript. All authors read and approved the final manuscript.
